# Communicative Skills Assessment Scale: Contributions to the Validation of Autism Spectrum Disorder

**DOI:** 10.1177/23969415251362626

**Published:** 2025-07-27

**Authors:** Diana Oliveira, Marisa Lousada, Daniela Figueiredo

**Affiliations:** 1 56062 Universidade de Aveiro–ESSUA, Aveiro, Portugal

**Keywords:** Autism Spectrum Disorder, scale, communication, reliability, validity

## Abstract

**Background and Aims:**

Speech–language pathologists actively participate in the assessment of pragmatics in children with Autism Spectrum Disorder (ASD). However, in Portugal, there is a shortage of valid and reliable instruments to assess this domain in detail. Therefore, it was deemed relevant to help validate the *Escala de Avaliação de Competências Comunicativas* (EAC) (Communicative Skills Assessment Scale) in this population.

**Methods:**

A cross-sectional, descriptive correlational study was conducted. The sample included 97 children, aged between 4 and 7 years and 11 months, diagnosed with ASD with European Portuguese as their native language. As an exclusion criterion, the co-occurrence of intellectual developmental disorder was defined. The internal consistency of the EAC was analyzed through the calculation of Cronbach's alpha and test–retest reliability through the calculation of the intraclass correlation coefficient. Convergent validity was also analyzed using Spearman's correlation coefficient.

**Results:**

The EAC presented a high internal consistency value, with a Cronbach's alpha of 0.956. The test–retest reliability analysis (n = 27) revealed good stability in the participants’ responses at both assessment moments, with ICC values varying between 0.642 and 0.842. In the study of convergent validity, statistically significant correlations were observed between the EAC total score and the two scales of the Strengths and Difficulties Questionnaire-Portuguese Version (SDQ-Por): relationship problems with colleagues (r_s_ = −0.273; *p* < .01) and prosocial behavior (r_s_ = 0.606; *p* < .01).

**Conclusion:**

The EAC presents good reliability and validity for children with ASD, suggesting its adequacy as a research and clinical tool for testing pragmatics within this population.

**Implications:**

This study will improve clinical decision making regarding ASD and guide future research.

## Introduction

According to 2020 data from the United States Centers for Disease Control and Prevention, one in every 36 children has been diagnosed with Autism Spectrum Disorder (ASD; Maenner et al., 2023), representing an increase of approximately 20% since 2018. Recent epidemiological studies conducted in Europe also support an increase in the prevalence of ASD (Bougeard et al., 2021).

Over the past four decades, the study of pragmatics has become increasingly relevant to both the assessment and the intervention of children with ASD (Fujiki & Brinton, 2017), since one of the main characteristics of these children is the inadequate use of pragmatic skills in various social contexts (Georgiou & Spanoudis, 2021).

Children with ASD may display difficulties in developing and maintaining social relationships and a lack of interest in various conversation topics (Hage et al., 2022). Some children develop oral language and demonstrate an interest in reading and writing at early stages, while others present a complete absence of speech (Barby et al., 2022). Conversely, there are children who, despite employing oral language, exhibit pronoun reversals, jargon, and echolalia (Lopes-Herrera et al., 2023).

Children with ASD also utilize altered prosody. Noticeable difficulties are observed, particularly in intonation and the emphasis used throughout their speech, which makes it sound “robotic” and stereotyped (Bishop et al., 2017).

In addition, figurative language is impaired, which may be associated with difficulties with the theory of mind (Deliens et al., 2018). Individuals with ASD have significant difficulties understanding irony because it is closely linked to interpreting different mental states (Collins et al., 2014). However, there is great variability within ASD, meaning that the pragmatic profile of one child may not be the same as another's (Pereira, 2019).

Internationally, several standardized tools have been designed to assess pragmatic skills in preschool-aged children, including the Language Use Inventory (LUI): An Assessment for Young Children's Pragmatic Language Development (O'Neill, 2007; 2009), the Children's Communication Checklist (CCC; Bishop, 1998), the Communication and Symbolic Behaviour Scales Developmental Profile (CSBS-DP; Wetherby & Prizant, 2002), the Social Skills Rating System (SSRS; Gresham & Elliott, 1990), the Clinical Evaluation of Language Fundamentals—Fourth Edition (CELF-4; Semel et al., 2006) and the Test of Problem Solving 3: Normative Update (TOPS-3: NU; Bowers et al., 2018). The CCC, SSRS, CELF-4, and the TOPS-3: NU can also be used with school-aged children (Bowers et al., 2018; Pereira, 2019; Pereira & Lousada, 2022; Seabra, 2021).

The LUI, a standardized parent-reported inventory, has proven effective at identifying children with typical language development and those with language impairments. Its internal consistency values range from 0.80 to 0.98 across 10 of the 15 subscales (O’Neill, 2007).

The CCC-2 (previously called ‘CCC’ before its revision) was originally validated in the United Kingdom with a sample of 542 individuals with typical development. The CCC-2 was reviewed by six researchers who collected data from German children and adolescents (ages 4–17) from four groups, including ASD (n = 195) and typical development (n = 417). This resulted in a simpler and shorter version, the CCC-R (Wellnitz et al., 2021). According to the authors, 31 of the 70 items from the CCC-2 were removed from the final CCC-R version due to limitations in the factor structure and psychometric properties. The CCC-R demonstrates high internal consistency (α = 0.96).

The CSBS-DP, a questionnaire completed by a parent or someone who interacts with a child daily, shows test–retest reliability values ranging from 0.64 to 0.91, calculated using a Pearson's correlation coefficient over a 4-month interval (Wetherby et al., 2002).

The SSRS enables the evaluation of problem behaviors, academic competence, and social skills through questionnaires completed by parents, teachers, and students. It demonstrates internal consistency for the social skills scale, with Cronbach's alpha values ranging from 0.83 to 0.94. Test–retest reliability for the social skills scale questionnaire was 0.85 for teachers and 0.68 for students (Gresham & Elliott, 1990).

The CELF-4 includes, among other components, a subtest called the Pragmatics Profile, which is presented in checklist format. The Cronbach's alpha coefficients for the subtests range from 0.69 to 0.91. Test–retest reliability coefficients ranged from 0.71 to 0.86 for the existing subtests. Inter-rater reliability varies from 0.88 to 0.99 for subtests involving clinical judgments and the interpretation of scoring rules (Semel et al., 2006).

The TOPS-3: NU displays good reliability (α = 0.82) and sensitivity and specificity levels of 0.69 and 0.89, respectively, distinguishing between children with and without language impairments (Bowers et al., 2018).

At school age, the following instruments are particularly noteworthy: the Clinical Evaluation of Language Fundamentals–Third Edition (CELF-3; Semel et al., 2000), Expression Reception and Recall of Narrative Instrument (ERRNI; Bishop, 2004), and the Assessment of Comprehension and Expression (ACE; Adams et al., 2001). We will discuss each in turn below.

The CELF-3 investigates receptive and expressive language skills. It presents internal consistency (values ranging from 0.83 to 0.95 for overall standard scores and from 0.54 to 0.95 for individual subtests). The test–retest reliability ranges from 0.80 to 0.91 for overall standard scores and from 0.52 to 0.90 for individual subtests. Inter-rater reliability ranged from 0.95 to 0.99 (Semel et al., 2000).

The ERRNI can be a valuable tool for gathering information on narrative abilities, which are tested by two stories. Regarding internal consistency, Cronbach's alpha values range between 0.75 and 0.90. Reliability scores for the Fish Story (initial narrative) are 0.86 and 0.90 and for the Beach Story, 0.85 and 0.90. Although normative data are available for children as young as 4 years old, the instrument is designed for use with children aged 6 years and older (Bishop, 2004).

The ACE investigates both verbal expression and language comprehension. Inter-rater reliability was not reported; however, test–retest reliability was 0.83. Cronbach's alpha, calculated from the narrative propositions, was 0.73 (Adams et al., 2001).

For European Portuguese (EP), the instruments available to assess pragmatics are the following Portuguese versions of the Language Use Inventory (LUI-PORTUGUÊS; Guimarães, 2013, 2012): the CCC (CCC-P; Mendes, 2011), the Pragmatic Profile of Daily Communicative Abilities in School-aged Children (Almeida & Rocha, 2009), the Inventory of Social Communication Skills (ICCS; Carvalho & Cruz-Santos, 2012), the Communication Scale for Educators and Teachers: Preschool Age Communication Scale (ECIPE)–Educator Version (Marinho, 2021), and the School Age Communication Scale (ECIE)—Teacher Version (Azevedo, 2021). However, none of these is validated for children with ASD.

The LUI-PORTUGUÊS has three parts: communication through gestures, communication through words, and communication through sentences (Guimarães et al., 2013). It aims to test children aged between 18 and 47 months. The instrument includes reliability studies, conducted through internal consistency and test–retest stability, content and construct validity, assessed through factor analysis. All three parts of the inventory illustrate good internal consistency, with an alpha value greater than 0.80 in a sample of 1,555 children. For the test–retest study, conducted with a 4-week interval, 34 participants were included. The correlations between the scores obtained at both time points were high, from 0.83 to 0.98 (Cruz-Santos & Guimarães, 2020).

The communication scale for both early childhood educators and teachers has reliable studies of reliability and validity. In the ECIPE and ECIE, very high Cronbach's alpha values were observed: 0.98 and 0.972, respectively. Furthermore, all items of the communication scale are homogeneous and accurately measure what they intend, both in children aged 5 and 6, as well as in children aged 6 to 9 years (Dias, 2021; Marinho, 2021).

The ICCS initially included three dimensions (communicative initiation and function, discourse management and the adaptation of discourse to the characteristics and needs of the interlocutor and context). However, 16 items were removed. Consequently, the final version of the inventory was reduced to 14 items based on the validity study and was reorganized into two dimensions: initiation and management (Carvalho, 2019). Following the validation study of the inventory, which involved 292 children, it was deemed a valid instrument. For example, satisfactory Cronbach's alpha values are observed in the two identified latent dimensions (initiation and management). In the initiation dimension, the alpha value was 0.90, while in the management dimension, the value was slightly lower—0.87 (Carvalho, 2019).

Recently, the Communicative Skills Assessment Scale (*Escala de Avaliação de Competências Comunicativas* [EAC]; Seabra et al., 2021) was developed to enable a detailed assessment of pragmatics validated in children with typical language development aged 4 to 8 years. The EAC aims to help identify alterations in pragmatic skills and shows favorable psychometric characteristics concerning both content validity and construct validity and reliability (internal consistency and test–retest reliability; Seabra, 2021). However, validation studies of EAC in children with ASD have not yet been conducted.

In Portugal, 90.2% (n = 83) of speech–language pathologists (SLPs) use informal assessments to evaluate pragmatic skills in ASD (Pereira et al., 2024). Considering the lack of properly validated instruments in this area for children aged 4 and older (Pereira, 2019; Seabra, 2021; Seabra et al., 2021), it was deemed highly relevant to contribute to the validation of the EAC for ASD, analyze internal consistency, verify test–retest reliability and evaluate the convergent validity of the scale.

CCC-2 has been translated into and culturally adapted for Brazilian Portuguese. The internal consistency of the instrument was also analyzed, with values ranging from 0.75 to 0.90 (Costa et al., 2013).

## Methods

### Design and Participants

A study with a cross-sectional, descriptive–correlational design was conducted. To be part of the study, participants met the following inclusion criteria: diagnosis of ASD, age range between 4 years and 11 months and Portuguese as their native language. Those with intellectual development disorders were excluded. Confirmation of an ASD diagnosis was provided by the parents through the completion of a questionnaire. In Portugal, a clinical diagnosis is provided by a pediatrician or psychiatrist (specialized in neurodevelopment), according to the criteria of the Diagnostic and Statistical Manual of Mental Disorders, Fifth Edition (DSM-V), Autism Diagnostic Observation Schedule (ADOS) and/or Autism Diagnostic Interview—Revised (ADI-R).

Initially, the population consisted of 134 participants, 37 of whom were excluded due to the following factors: suspected but undiagnosed ASD, Portuguese was not the native language, inappropriate age range and lack of consent to access data for the research study. Thus, 97 children participated in the study. Both verbal and nonverbal children were included.

### Data Collection Procedures

The Ethics Committee of the Health Sciences Research Unit at the Coimbra Nursing School (Approval No. 695/07-2020) greenlit the study. Subsequently, institutions working with children with neurodevelopmental disorders were contacted via email and phone to identify potential participants meeting the inclusion and exclusion criteria. All legal guardians of the children received an information sheet about the study to ensure that they understood the procedures and the study's motivations.

All participants initially completed the data collection protocol, which consisted of a sociodemographic questionnaire, the EAC and the Portuguese Version of the Strengths and Difficulties Questionnaire (SDQ-Por; Fleitlich et al., 2005), which was originally the Strengths and Difficulties Questionnaire (SDQ).

The sociodemographic questionnaire included information to characterize each child's profile, such as questions about their date of birth, gender, education, and the presence or absence of any associated conditions (e.g., attention deficit hyperactivity disorder—ADHD).

The EAC is a parent-reported inventory designed to help identify difficulties in pragmatic skills in children aged 4 to 8. The scale includes eight dimensions: (1) communicative intentions (14 items), (2) interaction skills (9 items), (3) response in communicative contexts (3 items), (4) comprehension in communicative contexts (4 items), (5) discourse coherence (5 items), (6) discourse cohesion (2 items), (7) comprehension and use of nonliteral language (6 items), and (8) paralinguistic aspects (2 items), totaling 45 items. Each item offers four response options: (1) never, (2) rarely, (3) sometimes, and (4) often, which should be selected based on the frequency with which the child displays the behavior described. Responses are based on the perceptions of parents or educators who choose the option that most closely matches the child's daily behavior. The maximum EAC score is 180, and the average completion time is 10 to 15 min (Seabra et al., 2021). The EAC scale has been validated for children with typical language development (Seabra, 2021). The EAC can be completed by parents, caregivers, educators, or teachers, with results interpreted by SLPs, psychologists, and pediatricians. The SDQ-Por aims to evaluate appropriate and inappropriate social behaviors in children and adolescents aged 4 to 16 across the following scales: emotional symptoms, behavioral problems, hyperactivity, peer relationship issues, and prosocial behavior (Goodman, 1997). Each scale includes five items, totaling 25 items, with three response options for each: “not true,” “somewhat true,” and “certainly true.” The average completion time for the questionnaire is 3 to 5 min.

The response option “somewhat true” always scores 1 point. However, the scoring for “not true” and “certainly true” varies, receiving either 0 or 2 points depending on the item (Goodman, 2001). For items 7 (“Obeys easily, usually does what the parents ask”), 11, 14, 21, and 25, the response “not true” scored a 2, and “certainly true” scored 0. These five items are reverse scored during data analysis. For the remaining 20 items (e.g., “Is restless, constantly on the move, never stays still”), the scores are 2 for “certainly true” and 0 for “not true.”

In the posterior phase of the study, a test–retest reliability analysis was deemed necessary. A subsample was selected and asked to complete a second form that included only select sociodemographic questions and the EAC.

### Statistical Analysis

The data were analyzed using descriptive and inferential statistics with the Statistical Package for the Social Sciences (IBM SPSS Statistics), Version 28.0 for Windows. Regarding the reliability study, internal consistency was evaluated by calculating Cronbach's alpha coefficient, with values interpreted as follows (George & Mallery, 2003): very good (α > 0.900), good (α > 0.800), acceptable (α > 0.700), questionable (α > 0.600), peak (α > 0.500), and unacceptable (α < 0.500; Wadkar et al., 2016). Subsequently, test–retest reliability analysis was conducted by calculating the intraclass correlation coefficient (ICC), with values ranging from 0 (indicating no correlation between results) to 1.0 (indicating perfect correlation). Values below 0.5 indicate poor reliability, while values above 0.9 indicate excellent reliability (Liljequist et al., 2019). The test–retest reliability analysis was performed with a subsample of participants who completed the EAC at two times at a 15-day interval, as recommended by the literature (Gignac, 2009).

The Spearman’s correlation coefficient was calculated to analyze the convergent validity between the EAC and the SDQ-Por. This choice was made because the Kolmogorov–Smirnov test indicated a *p*-value of < .05, meaning that the variables did not follow a normal distribution.

## Results

### Sample Characterization

The children (n = 97) were predominantly male (83.5%) and primarily attended preschool (79.4%). A significant majority were receiving treatment in speech and language therapy (95.9%) and had ADHD (22.7%) as a comorbidity. The sociodemographic and clinical characteristics of the sample are presented in Table 1.

**Table 1.** Sociodemographic and Clinical Characteristics of the Sample.

Variables	N	%
Relationship with the child		
Mother	91	93.8
Father	5	5.2
Speech–language pathologist	1	1.0
Age		
4–5 years	58	59.8
6–7 years	39	40.2
Gender		
Male	81	83.5
Female	16	16.5
Education		
Preschool	77	79.4
First and second grade	20	20.6
Does the child currently attend or did the child previously attend speech and language therapy?		
Yes	93	95.9
No	4	4.1
Does the child have ADHD?			
Yes	22	22.7
No	75	77.3
Does the child take medication?		
Yes	26	26.8
No	71	73.2

*Note*. ADHD=Attention Deficit Hyperactivity Disorder.

Of the remaining children (77.3%), only four were identified with the following associated conditions: strabismus, XYY syndrome, hyperlexia, and specific learning disorder.

According to the total number of children eligible for the study, 71 were not taking any medication. Among the 26 children who were on medication, 21 were receiving treatment for ADHD, with three taking first-line medications (methylphenidate), and 18 on second-line medications (risperidone). Of the remaining five children, three were prescribed Melamil Tripto, one was on Depakene, and the other was taking aripiprazole.

Notably, approximately 68% of the children in the sample displayed atypical scores on the “Hyperactivity” scale for the following items: “Is restless, very fidgety, never still”; “Can't settle down. Is always moving their legs or hands”; “Gets distracted easily, is always daydreaming”; “Can stop and think before acting”; and “Usually finishes what they start, pays good attention.” In addition, 43% of the children exhibited a borderline severity level in the total difficulty score (see Table 2).

**Table 2.** Frequency of SDQ-Por Dimensions.

	Mean	SD	Limits	Severity
SDQ Difficulties	17.21	5.90	0–40	Borderline
Emotional symptoms	2.79	2.13	0–10	Typical
Behavior problems	3.42	2.22	0–10	Typical
Hyperactivity	6.79	2.31	0–10	Atypical
Peer relationship problems	4.20	1.99	0–10	Borderline
Prosocial behavior capabilities	5.38	2.43	0–10	Borderline

*Note*. SDQ-Pro= Strengths and Difficulties Questionnaire–Portuguese Version.

The mean and standard deviation (SD) were also analyzed for each age group (in months), with the calculation of the independent samples t-test. It was possible to identify statistically significant differences (*p* = .049; t = 1.985) between the means of both age groups: 95.41 ± 19.3 (4A0M-5A11M) and 104.67 ± 26.0 (6A0M-7A11M), although the *p*-value is at the threshold of being nonsignificant.

### Reliability Analysis–Internal Consistency

Table 3 presents the value of α_del_ when the item was removed, the mean (M), the SD and the item–total correlation (ITC) values, showing that all the items of the EAC contributed significantly because they exhibited an α value > 0.900.

**Table 3.** Descriptive Results of the EAC (n = 97).

Item	M	SD	ITC	α_del_	Item	M	SD	ITC	α_del_
1. Eye contact	3.25	0.60	0.326	0.956	24. Respond when requested	2.84	0.94	0.599	0.955
2. Interpret facial expressions	3.38	0.71	0.425	0.956	25. Respond to a greeting	3.21	0.88	0.519	0.955
3. Use facial expressions	3.34	0.72	0.414	0.956	26. Negotiate with the other	2.39	1.16	0.674	0.955
4. Use gestures	3.29	0.83	0.303	0.956	27. Understand the other person's intentions	2.51	0.93	0.472	0.956
5. Interpret gestures	2.40	0.93	0.468	0.956	28. Understand emotional states	2.93	0.82	0.381	0.956
6. Make requests	3.05	1.07	0.700	0.954	29. Understand direct requests	3.49	0.68	0.283	0.956
7. Get the other person's attention	3.49	0.79	0.391	0.956	30. Understand indirect requests	2.16	1.03	0.389	0.956
8. Take the initiative	1.96	1.04	0.676	0.954	31. Choose an appropriate topic	1.72	0.85	0.752	0.954
9. Express what they feel	2.59	0.93	0.630	0.955	32. Relate events in sequence	1.88	0.99	0.731	0.954
10. Request clarification	1.80	0.99	0.660	0.955	33. Attend to whether the other person already has the information	1.37	0.56	0.647	0.955
11. Narrate what they experienced	1.92	1.04	0.699	0.954	34. Do not repeat the same topics	1.75	0.92	0.611	0.955
12. Narrate what they want to experience	1.89	0.99	0.706	0.954	35. Adapt the information to the situation	1.77	0.87	0.730	0.954
13. Give instructions	1.65	0.89	0.669	0.955	36. Understand deictics	2.77	0.91	0.484	0.956
14. Praise the other	1.87	1.03	0.746	0.954	37. Use deictics	2.36	1.01	0.624	0.955
15. Attend to a conversation	2.34	0.79	0.508	0.955	38. Use idiomatic expressions	1.21	0.58	0.355	0.956
16. Initiate a conversation	2.26	1.03	0.742	0.954	39. Understand idiomatic expressions	1.20	0.51	0.380	0.956
17. Participate in a conversation	2.07	0.96	0.786	0.954	40. Understand irony	1.27	0.57	0.383	0.956
18. Change the subject of the conversation	1.63	0.83	0.758	0.954	41. Use irony	1.19	0.46	0.421	0.956
19. End a conversation	1.63	0.91	0.723	0.954	42. Tell jokes	1.24	0.54	0.591	0.955
20. Adapt vocabulary to the interlocutor	1.69	0.99	0.637	0.955	43. Understand jokes	1.35	0.63	0.660	0.955
21. Adapt vocabulary to the context	1.65	0.91	0.561	0.955	44. Interpret tone of voice	3.18	0.84	0.217	0.957
22. Adapt the amount of information	1.74	0.88	0.578	0955	45. Modulate tone of voice	2.93	1.04	0.450	0.956
23. Contextualize the interlocutor	1.46	0.68	0.581	0.955					

*Note*. EAC= Escala de Avaliação de Competências Comunicativas; ITC= item–total correlation.

Table 4 indicates that the internal consistency was very good for the total EAC (α = 0.956) and good for the eight dimensions, with α values < 0.70 observed in two dimensions: “IV. Understanding in communicative contexts” and “VIII. Paralinguistic aspects.”

**Table 4.** Internal Consistency for Each Dimension and the Total EAC (n = 97).

Dimension	Number of items	M	SD	Alpha
I. Communicative intentions	14	35.88	8.13	0.889
II. Interaction skills	9	16.47	5.83	0.887
III. Response in communicative contexts	3	8.43	2.39	0.710
IV. Comprehension in communicative contexts	4	11.09	2.27	0.548
V. Discourse coherence	5	8.49	3.49	0.876
VI. Discourse cohesion	2	5.13	1.79	0.853
VII. Comprehension and use of nonliteral language	6	7.44	2.39	0.818
VIII. Paralinguistic aspects	2	6.10	1.62	0.639
EAC (Total)	45	99.05	22.81	0.956

*Note*. EAC= Escala de Avaliação de Competências Comunicativas.

Considering the results obtained in the domain “IV. Understanding in communicative contexts” and “VIII. Paralinguistic aspects,” it was deemed important to analyze the value of α_del_ as if the item were removed, along with the mean (M), the SD and the ITC. The values obtained are described in Table 5.

**Table 5.** Descriptive Results of Dimensions IV and VIII of the EAC (n = 97).

Item IV	M	SD	ITC	α_del_	Item VIII	M	SD	ITC	α_del_
27. Understand the other person's intention	2.51	0.93	0.291	0.514	44. Interpret tone of voice	3.18	0.84	0.477	
28. Understand emotional states	2.93	0.82	0.343	0.469	45. Modulate tone of voice	2.93	1.04	0.477	
29. Understand direct requests	3.49	0.68	0.413	0.435					
30. Understand indirect requests	2.16	1.03	0.325	0.493					

*Note*. EAC= Escala de Avaliação de Competências Comunicativas; ITC= item–total correlation.

The Cronbach's alpha value in the dimension “IV. Understanding in communicative contexts” was 0.548. When the item in the dimension was removed, the value of α_del_ did not increase. In the dimension “VIII. Paralinguistic aspects,” it was impossible to analyze the value of α_del_ because this dimension comprises only two items.

### Reliability Analysis—Test–Retest

In the second phase, the test–retest reliability analysis was conducted with a subsample of 27 children, predominantly male (88.9%), who were attending preschool (74.1%). The sociodemographic characterization of the subsample is in Table 6.

**Table 6.** Sociodemographic Characterization of the Subsample.

Variables	n	%
Relationship with the child		
Mother	25	92.6
Father	2	7.4
Age		
4–5 years	14	51.9
6–7 years	13	48.1
Gender		
Male	24	88.9
Female	3	11.1
Education		
Preschool	20	74.1
First grade	7	25.9

Table 7 presents the correlation values between the scores obtained at the two time points, ranging from 0.642 to 0.961. However, the range attained between the lower and the upper limits was high, particularly in dimensions IV, VI, and VIII, indicating that the evaluation scores given by the participants varied more than expected.

**Table 7.** Mean, Standard Deviation, and Cronbach's Alpha for the Test and Retest Phases, ICC and CI.

Dimension	Test			Retest			ICC	CI 95%	
	M	SD	Alpha	M	SD	Alpha		Lower limit	Upper limit
I. Communicative intentions	34.44	7.85	0.888	35.19	7.42	0.879	0.961	0.916	0.982
II. Interaction skills	16.56	6.05	0.930	16.15	6.33	0.953	0.947	0.884	0.976
III. Response in communicative contexts	7.81	2.68	0.829	8.33	2.45	0.843	0.906	0.776	0.954
IV. Comprehension in communicative contexts	10.52	2.33	0.686	10.37	2.44	0.748	0.642	0.222	0.842
V. Discourse coherence	8.15	2.90	0.903	8.22	3.51	0.923	0.905	0.797	0.958
VI. Discourse cohesion	5.11	1.67	0.852	5.30	1.77	0.853	0.738	0.434	0.883
VII. Comprehension and use of nonliteral language	7.85	2.63	0.850	7.37	2.37	0.921	0.897	0.762	0.951
VIII. Paralinguistic aspects	5.70	1.41	0.542	6.11	1.53	0.786	0.728	0.416	0.875

*Note*. CI=confidence interval; ICC= intraclass correlation coefficient.

### Analysis of Convergent Validity

The analysis of convergent validity conducted between the EAC and the SDQ-Por attempted to verify whether there was a correlation between the two instruments. As mentioned, children with ASD tend to have a high probability of revealing social difficulties among their peers (Adachi et al., 2018).

As displayed in Table 8, there were statistically significant correlations between the EAC and the SDQ. For example, a negative and statistically significant correlation was observed (r_s_ = –0.201; *p* < .05) between the total score of difficulties on the SDQ-Por (emotional symptoms, conduct problems, hyperactivity, and peer relationship problems) and Dimension IV. Understanding in communicative contexts of the EAC. In other words, the greater the difficulties reported in the SDQ, the lower the level of understanding in communicative contexts among children with ASD.

**Table 8.** Spearman Correlation Coefficient Between the EAC and the SDQ-Por.

	SDQ-Por					
	Difficulties					Capabilities
	Difficulties (total)	Emotional symptoms	Behavior problems	Hyperactivity	Peer relationship problems	Prosocial behavior
EAC						
EAC (total)	−0.095	0.123	−0.027	−0.139	**−**.273**	.606**
Communicative intentions	−0.049	0.098	0.018	−0.059	**−**.224*	.546**
Interaction skills	−0.080	0.161	−0.038	−0.181	**−**.238*	.539**
Response in communicative contexts	−0.088	0.096	−0.011	−0.109	**−**.272**	.506**
Comprehension in communicative contexts	**−**.201*	−0.043	−0.019	−0.169	**−**.353**	.472**
Discourse coherence	−0.065	0.161	−0.050	−0.124	**−**.201*	.528**
Discourse cohesion	−0.070	0.112	−0.010	−0.183	−0.152	.364**
Comprehension and use of nonliteral language	−0.031	−0.018	−0.012	−0.052	−0.035	.378**
Paralinguistic aspects	−0.052	0.122	−0.074	−0.079	−0.128	.354**

*Note*. EAC= Escala de Avaliação de Competências Comunicativas; SDQ-Pro= Strengths and Difficulties Questionnaire–Portuguese Version.

**The correlation is significant at the level of 0.01 (bilateral).

*The correlation is significant at the level of 0.05 (bilateral).

A negative and statistically significant correlation was also observed (r_s_ = –0.273; *p* < .01) between the score on the Peer Relationship Problems Scale (difficulty) and the total score of the EAC, as well as between the score on the Peer Relationship Problems Scale and 5 of 8 EAC dimensions: I. Communicative intentions (r_s_ = –0.224; *p* < .05); II. Interaction skills (r_s_ = –0.238; *p* < .05); III. Response in communicative contexts (r_s_ = –0.272; *p* < .01); IV. Understanding in communicative contexts (r_s_ = –0.353; *p* < .01), and V. Coherence in discourse (r_s_ = –0.201; *p* < .05).

In contrast, a positive and statistically significant correlation was observed (r_s_ = 0.606; *p* < .01) between the score on the Prosocial Behaviour Scale (ability) and the total score of the EAC, as well as between the Prosocial Behaviour Scale and all dimensions of the EAC. This suggests that the higher the frequency of the Prosocial Behaviour Scale, the better the pragmatic comprehension and expression skills demonstrated by the children.

## Discussion

The main objective of this study was to contribute to the validation of the EAC in children with ASD aged between 4 and 8 years. A reliability study was conducted through the collection and analysis of data in terms of internal consistency, based on a sample of 97 children, and test–retest reliability, using a subsample of 27 children. Convergent validity was determined by correlating the EAC with the SDQ-Por.

It was possible to observe statistically significant differences between the means of both age groups: 95.41 ± 19.3 (4A0M–5A11M) and 104.67 ± 26.0 (6A0M-7A11 M), as was the case in Seabra (2021): 158.05 ± 13.1 (4A0M-5A11M) and 163.51 ± 13.5 (6A0M-8A0M) with typically developing children. However, in the present study, the results obtained in both age groups were much lower than those of Seabra (2021) due to the clinical diagnosis (ASD) presented by the children.

The 45 items of the EAC showed an α value > 0.900, indicating that even if an item was removed, the alpha value did not change, meaning that all items contributed significantly. Furthermore, the internal consistency was very good for the total EAC (α = 0.956) and mostly good across all dimensions, except two: IV. Understanding communicative contexts and VIII. Paralinguistic aspects, where an α value < 0.70 was evident (Wadkar et al., 2016). In the initial version of the EAC, the results were similar: α = 0.929 for the total EAC and α < 0.70 in dimensions IV and VIII (Seabra, 2021). The values presented are also comparable to those achieved in international instruments, such as the CCC-R (α = 0.96) and TOPS-3 (α = 0.82; Wellnitz et al., 2021; Bowers et al., 2018).

Concerning the evaluation of test–retest reliability, it was possible to observe good stability in the results obtained at both assessment time points, as the correlations between the scores ranged from 0.642 to 0.961. The EAC values for children with typical development illustrated even greater stability, ranging from 0.94 to 1.00 (Seabra, 2021). However, in Bishop, the values obtained through the application of the CCC-2 (United States version) in a sample with ASD ranged from 0.86 to 0.96, similar to those of the present study (Bishop, 2006). The results obtained from the calculation of the confidence interval demonstrate that in the dimensions IV. Understanding in communicative contexts; VI. Coherence in discourse; and VIII. Paralinguistic aspects, the participants’ evaluations varied considerably. For example, dimension IV obtained limits from 0.222 (lower limit) to 0.842 (upper limit). However, the values among the evaluations of participants in the research study for the population without language development impairments remained very close, considering, for example, Domain IV: 0.940 (lower limit) and 0.989 (upper limit; Seabra et al., 2021). The differences found in both studies may relate to the fact that children with pragmatic difficulties may not exhibit as consistent behavior as children with typical development.

Regarding the analysis of convergent validity between the EAC and the SDQ-Por, it was observed that the Peer Relationship Problems Scale was negatively correlated with the total EAC score, while the Prosocial Behaviour Scale was positively correlated with the total EAC score. This indicates that children with better scores on the EAC exhibit fewer behavior problems and better prosocial behavior because they tend to have a higher likelihood of presenting social difficulties among peers (Weismer, 2013).

Children who have difficulty initiating, maintaining, and concluding a conversation, who do not respond to their peers or acknowledge them and who do not understand the requests or intentions of others when they want to communicate with them have shown impairment in their relationships with peers (negative correlation with the Peer Relationship Problems Scale). It was found that these children tend to isolate themselves more, relate better to adults than to other children and are sometimes targets of bullying behavior from their peers.

However, the Prosocial Behaviour Scale correlated positively with all eight dimensions of the EAC. When a child can praise others, interpret the facial expressions and gestures used by other children, understand the emotional states of their peers and interpret the tone of voice of others (e.g., when someone is angry), she can exhibit appropriate and kind behavior toward others. This includes enjoying helping peers, teachers, and educators; sharing toys and school supplies and being sensitive to the feelings of others. In summary, the greater the impairment in language use, the more significant the problems in peer relationships will be and vice versa (Weismer, 2013).

A limitation of the study is the small size of the subsample (n = 27) for the test–retest analysis, considering the total sample (n = 97). Thus, further research with larger samples is needed to explore other psychometric characteristics, such as sensitivity and specificity.

In addition, the researcher was not present during the completion of the data collection protocol to address any potential questions or uncertainties that the informants might have had. While participants were provided with the researcher's contact information for clarification, the absence of direct interaction could have influenced the quality or accuracy of the data.

## Conclusion

The results demonstrated that the EAC had good reliability characteristics, particularly regarding the internal consistency of each item and the overall stability of the test–retest analysis.

Regarding the convergence analysis, statistically significant correlations were also observed between the total EAC score and two scales of the SDQ-Por: Peer Relationship Problems and Prosocial Behaviour. This demonstrates that children with low scores on the EAC exhibit more behavior problems and poorer performance in prosocial behavior.

Thus, this work reinforces that the EAC is a valuable tool for testing pragmatic skills in children aged between 4 and 8 years, with practical applications for both clinical and educational settings. Its use can facilitate the early identification of social communication difficulties, inform individualized intervention planning and support ongoing process monitoring. Given its significant correlations with behavioral indicators, the EAC also holds promise as a tool for broader developmental screening.

Future researchers should focus on validating the EAC with larger and more diverse populations, analyzing its sensitivity to change over time and exploring adaptations such as digital versions to enhance accessibility and applicability across contexts.
